# Intentional Use of Voluntarily Fortified Foods in Relation to Socio-Demographic and Lifestyle Factors, Including Adherence to a DASH-Style Diet, Among University Students

**DOI:** 10.3390/nu18193188

**Published:** 2026-09-26

**Authors:** Ewa Sicińska, Dawid Madej, Maria Karolina Szmidt, Justyna Czerniak, Joanna Kaluza

**Affiliations:** Department of Human Nutrition, Institute of Human Nutrition Sciences, Warsaw University of Life Sciences (WULS-SGGW), Nowoursynowska 166, 02-787 Warsaw, Poland; dawid_madej@sggw.edu.pl (D.M.); maria_szmidt@sggw.edu.pl (M.K.S.); justyna_czerniak@sggw.edu.pl (J.C.); joanna_kaluza@sggw.edu.pl (J.K.)

**Keywords:** determinants, diet quality, fortification, food choice, young adults

## Abstract

**Background:** Voluntary food fortification is a common market-driven practice to improve nutritional value, but consumption of such products may also be associated with less healthy dietary patterns. This study aimed to identify socio-demographic and lifestyle factors, including diet quality, associated with the intentional use of voluntarily fortified foods (Intentional-VFFs) among university students. **Methods:** The cross-sectional study was conducted among 2123 university students in Warsaw. Data were collected through a self-administered health and lifestyle questionnaire and a 5-day food record. Multivariate logistic regression was used to estimate odds ratios (ORs) and 95% confidence intervals (CIs) to identify factors associated with Intentional-VFF use. Diet quality was assessed using a Dietary Approaches to Stop Hypertension (DASH) score. **Results:** Intentional-VFF use was reported by 44.3% of respondents and was significantly associated with age (each 1-year increment was associated with a 6% lower use), sex (male vs. female; OR: 0.63, 95%CI: 0.49–0.81), BMI (<18.5 vs. 18.5–24.9 kg/m^2^; OR: 0.63, 95%CI: 0.43–0.92), self-assessed nutritional knowledge (high vs. low; OR: 1.70, 95%CI: 1.20–2.42), excluding/including some products (yes vs. no; OR: 2.09, 95%CI: 1.55–2.83), and use of dietary supplements (OR: 1.62, 95%CI: 1.30–2.01). However, significant effect modification was observed; high nutritional knowledge was associated with Intentional-VFF use among men but not among women, whereas age was inversely associated with VFF use only among dietary supplement non-users. Intentional-VFF use was not significantly associated with the DASH score; however, each 50 g increment in vegetable consumption was associated with a 4% lower odds of VFF use (*p* = 0.013). **Conclusions:** Various factors, including sex, dietary supplement use, and nutritional knowledge, may be associated with Intentional-VFF use across different adult subgroups; however, establishing a link between their use and diet quality warrants further investigation.

## 1. Introduction

According to the World Health Organization, food fortification is the addition of essential nutrients to food (whether naturally present or not) to prevent or correct nutrient deficiencies in the general population or specific groups. Additionally, vitamins and minerals may be added to compensate for losses that occur during food processing (e.g., the addition of vitamin C to juices) or to ensure that substitute products provide a nutritional value comparable to the original foods they are intended to replace (e.g., the addition of calcium to plant-based beverages) [1].

Mandatory fortification is implemented when governments require food producers by law to add specific micronutrients to certain foods or food categories [1]. This approach has been used for decades in both Western and developing countries to address specific health issues. Its examples include iodised salt to prevent goitre, cereals enriched with B vitamins and iron to combat anaemia, and folic acid-fortified cereals to lower the risk of neural tube defects during pregnancy [2]. Voluntary fortification, also known as market-driven fortification, occurs when food manufacturers voluntarily add nutrients to products to boost their nutritional value and market appeal. It is a standard industry practice often used as a marketing strategy to remain competitive and differentiate products. In a given country, the decision between mandatory and voluntary fortification depends on several key factors, including the severity of the public health concern, the size and capacity of the relevant food industry, public awareness of nutritional needs, the political and regulatory context, and prevailing dietary habits [1].

The global market for voluntarily fortified foods (VFFs) is growing, offering products that may help reduce nutritional deficiencies. However, the law and regulations do not affect their raw composition. As a result, while VFFs can increase micronutrient intake, they may also contribute to higher consumption of fat, sugar, salt, and energy—components that, when consumed regularly over time, can increase the risk of hypertension and subsequently cardiovascular disease [3]. Moreover, the widespread availability of VFFs may reduce consumers’ motivation to regularly choose healthy foods, thereby contributing to lower overall diet quality. Consumers’ food choices depend on factors such as age, sex, income, educational level, traditions, beliefs, sensory preferences, lifestyle, as well as marketing strategies and the availability of product information [4].

Although VFFs in the European Union are regulated by uniform legislation [5], fortification practices and consumption patterns differ substantially across countries [6]. These differences reflect not only national nutrition policies and market dynamics but also cultural and dietary habits. Most existing studies on VFFs generalise their findings to broad age categories, often failing to capture population-specific factors associated with their use [3,7,8,9]. In contrast, our previous research identified some notable differences in the factors related to VFF use across different age groups, particularly between children and school-aged adolescents [10]. Among children, key factors included age, the number of meals consumed, and the presence of chronic conditions, whereas among adolescents, socio-economic status and physical activity level were influential. Despite these insights, there remains a notable lack of research on VFF consumption among young adults—a population actively developing its dietary habits and frequently targeted by the food industry through social media [11,12]. Understanding their motivations, behaviours, and socio-demographic background is therefore essential for assessing the public health implications of VFF consumption. To address this gap, this study aimed to determine the socio-demographic and lifestyle factors associated with intentional VFF use (Intentional-VFF) among university students, including those related to diet quality. Using the Dietary Approaches to Stop Hypertension (DASH) score as a measure of diet quality, we investigated whether higher diet quality is associated with a greater likelihood of Intentional-VFF use.

## 2. Materials and Methods

### 2.1. Study Design

This cross-sectional study was conducted from 2017 to 2022 among students enrolled in public universities in Warsaw, Poland. Participants were recruited through advertisements posted at university campuses and on social media platforms. The inclusion criteria for the study were as follows: age between 18 and 35 years and enrolment as students at public universities. Individuals were excluded if they had a medical condition requiring specialised dietary treatment or if their questionnaires were incomplete or inaccurately completed. Pregnant and breastfeeding women were also excluded.

The study protocol was approved by the Ethics Committee at the Faculty of Human Nutrition and Consumer Sciences, Warsaw University of Life Sciences (approval No. 09_1/2016). The study was conducted in accordance with the Helsinki Declaration. Written informed consent was obtained from each participant prior to their involvement in the study.

### 2.2. Data Collection

Data on socio-demographic and lifestyle factors were collected using a self-administered health and lifestyle questionnaire. Data on usual food intake were collected using a 5-day dietary record method. Individualised meetings were conducted with each participant, involving a qualified nutritionist or dietician, on two occasions: once during the recruitment meeting and again upon the return of the completed questionnaires. This approach facilitated a comprehensive understanding of the respondents’ dietary practices.

A total of 2202 students were enrolled in the study. Participants who did not meet the age inclusion criteria (*n* = 32), were pregnant or lactating (*n* = 9), or had medical conditions requiring dietary treatment (*n* = 6) were excluded. Moreover, students who failed to complete the health and lifestyle questionnaire or completed it only partially (*n* = 32) were excluded from the final study population. The analysis of factors associated with VFF use covered 2123 participants (Figure 1, Analysis 1).

### 2.3. Questionnaire Data

The health and lifestyle questionnaire was developed by the Department of Human Nutrition (WULS-SGGW) for an adult population, adapted to different age groups, and verified in an earlier study conducted with students; the research methodology has been presented in detail previously [13,14]. Briefly, the questionnaire comprised 31 questions across the following sections: socio-demographic characteristics, health and lifestyle status, nutritional knowledge and eating habits, and the use of dietary supplements and VFFs. At the beginning of the VFF section, a definition of VFFs and examples of such products were provided. VFFs were defined as food products labelled as containing added nutrients, which are voluntarily introduced by producers at the product manufacturing stage to enhance their nutritional value (e.g., breakfast cereals with added vitamins, yoghurt enriched with calcium and vitamin D). Respondents indicated whether their habitual diet included these products by selecting ‘Yes’, ‘No’, or ‘I don’t know’. Participants who selected ‘I don’t know’ were categorised into the *Unaware group*, whereas an Intentional-VFF user was defined as an individual who consciously reported VFF use in the questionnaire. Respondents who reported VFF use were prompted to select one or more predefined reasons for their usage, and also had the opportunity to provide additional, self-defined reasons. Conversely, respondents who indicated they do not use VFF were similarly asked to identify one or more predefined reasons for their non-use, with the option to elaborate on their own rationale. Respondents who declared that they used VFFs in their diet were asked about their sources of knowledge about these products. Both VFF users and non-users were asked whether they considered the product’s fortification when making their purchase.

### 2.4. Food Consumption Data

Food consumption was assessed using the 5-day food record method, covering four non-consecutive weekdays and one weekend day (Saturday or Sunday). Regarding data completeness, 57% of respondents completed all 5 days of the record, while 40% and 3% provided 3-day and 4-day records, respectively. During the recruitment meeting, participants were instructed to record all food and drink consumed on the specified days. Portion sizes were determined using kitchen scales or household measurements. Within three weeks of the recruitment meeting, respondents returned their food record questionnaires, which were then verified for accuracy and completeness. Among the 1904 respondents who returned the food record questionnaires, we excluded individuals who inaccurately or incompletely documented their food intake. Specifically, we excluded those who recorded data for fewer than 3 days (*n* = 8) and those with implausible energy intake values, defined as values outside ±3 standard deviations of the log-transformed energy mean, calculated separately for men and women (*n* = 8). The resulting acceptable ranges were 806–3347 kcal/day for women and 999–5028 kcal/day for men. This population-specific criterion is an established approach in nutritional epidemiology [15,16,17], as it accounts for the typically right-skewed distribution of self-reported dietary data and is preferred over arbitrary absolute caloric cut-offs for identifying extreme values. In the analysis of consumption details, including the types of fortified products and their contribution to overall dietary micronutrient intake, 1888 participants were included (Figure 1, Analysis 2).

The software of the National Institute of Public Health PZH–National Research Institute, which contains Polish tables of composition and nutritional value [18], was used to convert food intake data into energy and nutrient values. This database contains the most common products on the Polish market that contain added micronutrients. To classify VFFs reported in the food records, we applied a three-step procedure: (1) identification of all commercially processed food items reported in the records; (2) cross-referencing each item with its commercial ingredient list and nutrition declaration valid at the time of consumption, using manufacturer data and the national database; and (3) assigning the product to the VFF category only if the ingredient list explicitly indicated the voluntary addition of at least one micronutrient. VFFs not available in the database were matched to items with the most similar nutritional composition to minimise exposure misclassification. Based on the portion size of the fortified products consumed by each respondent, the quantities of micronutrients supplied with these products were calculated. Mandatory iodine-fortified salt and spreadable fats that are required to be fortified with vitamins A and D were not included in the analysis [19]. Based on the food record data, a Food-Record-VFF consumer was defined as an individual who reported at least one specified food item during the recording days. To assess the agreement between Intentional-VFF and Food-Record-VFF use, a cross-tabulation was performed for participants with available data from both methods (Supplementary Table S2).

### 2.5. Adherence to a DASH-Style Diet

Given that some fortified food products may have an unfavourable nutritional profile (e.g., high levels of fat, sugar, and salt) and may contribute to an increased risk of chronic diseases, including hypertension, diet quality was evaluated using the DASH score proposed by Fung et al. [20], with a modification to the sodium component. Sodium intake was energy-adjusted to 1000 kcal/day and included sodium from food products and prepared meals only, excluding salt added by respondents at the table. For each DASH component, respondents were classified into quintiles based on their intake ranking, separately for men and women. This sex-specific categorisation accounts for natural physiological differences in overall energy requirements and absolute food consumption. For products whose high consumption is considered beneficial (vegetables; fruits; legumes, seeds and nuts; wholegrains; and low-fat dairy products), the highest quintile (Q5) had the best score = 4, and the lowest (Q1) received the score = 0. For components considered unfavourable in excess (sodium; red and processed meats; and sweetened beverages), the lowest quintile (Q1) had the highest score = 4, and the highest quintile (Q5) = 0 (Supplementary Table S1). The total DASH score ranged from 0 (lowest relative adherence) to 32 (highest adherence to the DASH-style diet).

### 2.6. Statistical Analysis

The characteristics of Intentional-VFF users and non-users were compared using the Chi-squared test for categorical variables and the Mann–Whitney U test for quantitative variables, as the data did not follow a normal distribution. Micronutrient intake distributions were represented as mean with standard deviation and/or percentiles (5th, 50th, and 95th) for VFF consumption.

To examine the potential variables associated with Intentional-VFF use, logistic regression models were used to calculate odds ratios (ORs) with 95% confidence intervals (CIs). VFF non-users served as the reference category in all logistic regression analyses. The analysis was restricted to participants who intentionally included VFFs in their diet; respondents who were unaware of or unsure about the use of VFFs were excluded from the statistical analyses. Comprehensive model diagnostics, including assessments of multicollinearity, goodness of fit, discrimination, influential observations, residuals, and potential interaction effects, were performed to evaluate model adequacy.

The multivariate-adjusted models examined several potential socio-demographic and lifestyle factors that may be associated with Intentional-VFF use. These factors included age (continuous variable), sex (female, male), place of residence (rural area, small town, or city), socio-economic status (very good/good, or average/poor), physical activity (low, moderate, or high), body mass index (<18.5, 18.5–24.9, or ≥25 kg/m^2^), self-reported health status (at least good, or average/poor), cigarette smoking (no, yes), self-assessed nutritional knowledge (low, average, or high), health-oriented dietary modification (no, exclusion or inclusion of certain foods, or simultaneously exclusion and inclusion of certain foods), dietary supplement use (no, yes). Missing data on socio-demographic and lifestyle variables were modelled as separate categories.

Sensitivity analyses were performed, including a complete-case analysis, an analysis excluding participants surveyed online during the COVID-19 lockdown, and an analysis adjusting for recruitment year. In a further sensitivity analysis, participants who were uncertain about their Intentional-VFF use but classified as Food-Record-VFF consumers were reclassified as VFF users, and the regression analysis was repeated using this alternative classification. Additionally, potential effect modification was evaluated by including interaction terms for sex × nutritional knowledge and dietary supplement use × age. Although these interaction analyses were exploratory, their selection was guided by a priori hypotheses regarding potential effect modification. Specifically, we hypothesised that sex might modify the association between nutritional knowledge and VFF use, given well-documented sex-related differences in nutritional knowledge, dietary behaviours, food choices, and health-oriented dietary practices [21,22]. We also hypothesised that age might modify the relationship between dietary supplement use and VFF use, reflecting age-related variations in physiological needs, supplement use, and dietary behaviours [23,24]. Interaction terms were evaluated using likelihood ratio tests, and significant interactions were further examined via stratified analyses.

Furthermore, a multivariate logistic regression analysis was performed to determine the relationship between Intentional-VFF use and the DASH score and its individual components. The DASH score and its components were categorised into five groups using quintiles as cut-off points, with the first quintile serving as the reference group. The model was adjusted for those socio-demographic and lifestyle variables that were statistically significant in the multivariable-adjusted model of Intentional-VFF use.

The statistical analyses were performed using STATISTICA v. 13 (TIBCO Software, Inc., San Ramon, CA, USA), and *p*-values ≤0.05 were considered statistically significant.

## 3. Results

### 3.1. Characteristics of the Study Population

VFFs were incorporated into the habitual diet of 44.3% of respondents, while 22.3% were unaware or unsure whether they used such products. Socio-demographic and lifestyle characteristics of participants, stratified by Intentional-VFF use, are presented in Table 1. Compared with non-users, VFF users were younger (mean age: 21.8 ± 2.2 vs. 22.2 ± 2.3 years, *p* < 0.001) and comprised a higher proportion of women (78.0% vs. 65.4%). Furthermore, VFF users were significantly more likely than non-users to rate their nutritional knowledge as high (47.3% vs. 38.4%), to report health-oriented dietary modification (55.9% vs. 39.9%), and to use dietary supplements (51.3% vs. 34.3%).

The agreement between Intentional-VFF use and Food-Record-VFF consumption among participants is presented in Supplementary Table S2. Overall, both methods consistently classified 20.3% of participants as VFF users and 21.4% as non-users. Importantly, Food-Record-VFF consumption was also identified among 36.9% of participants who were uncertain about their VFF use in the *Health and lifestyle questionnaire* (8.1% of total participants). This highlights a discrepancy between self-reported intentional inclusion of these products in the diet and their consumption, indicating that uncertainty about VFF use does not necessarily imply non-consumption.

### 3.2. Reason for Intentional-VFF Use or Non-Use

During purchasing, a significantly higher percentage of Intentional-VFF users compared to non-users paid attention to whether the product was fortified (60.1% vs. 15.0%; *p* < 0.001) (Table 2). Participants cited beneficial health effects (71.8%) and insufficient dietary nutrients (29.7%) as the main reasons for using these products. The primary sources of information about VFFs were TV, radio, and websites (46.0%), as well as food labels (25.4%). The main reasons participants did not use VFFs were concerns about proper nutrition and no need for such products (48.0%), as well as not having heard of them (14.9%).

### 3.3. Socio-Demographic and Lifestyle Factors Associated with Intentional-VFF Use

Table 3 presents the multivariable-adjusted ORs for Intentional-VFF use by socio-demographic and lifestyle factors. An inverse, statistically significant association was observed between respondents’ age and Intentional-VFF usage. Each 1-year increment in age was associated with 6% (95% CI: 2–11%; *p* = 0.006) lower odds of Intentional-VFF use. Intentional-VFF users were less likely to be male (OR: 0.63, 95% CI: 0.49–0.81). Students with a BMI indicating underweight were less likely to use fortified products compared to those with a normal BMI (<18.5 vs. 18.5–24.9 kg/m^2^; OR: 0.63, 95% CI: 0.43–0.92). Compared to respondents who assessed their knowledge as low, those who assessed their nutritional knowledge as average or high were more likely to use Intentional-VFFs (OR: 1.53, 95% CI: 1.09–2.14, and OR: 1.70, 95% CI: 1.20–2.42, respectively). Among university students who deliberately modified their diet by including or excluding some food items, the odds of using Intentional-VFFs were 1.28-fold (95% CI: 1.01–1.63) higher, and among those who simultaneously included and excluded food products, the odds were 2.09-fold (95% CI: 1.55–2.83) higher. Respondents who used dietary supplements had 1.6-fold (OR: 1.62, 95% CI: 1.30–2.01) higher odds of using Intentional-VFFs compared to non-users. Diagnostic evaluation of the main logistic regression model confirmed adequate goodness-of-fit (Hosmer–Lemeshow χ^2^ = 8.23, *p* = 0.412), moderate discriminatory ability (AUC = 0.659), and the absence of multicollinearity or highly influential observations (Supplementary Table S3).

Sensitivity analyses yielded results consistent with the main analysis, showing no substantial changes in the observed associations. This was true when using a complete-case dataset (Supplementary Table S4), when excluding participants whose data were collected online during the COVID-19 lockdown, as well as when adjusting for recruitment year (Supplementary Table S5). Furthermore, reclassifying participants who were uncertain about their VFF use but identified as Food-Record-VFF consumers as Intentional-VFF users yielded similar results, except that self-assessed nutritional knowledge was no longer statistically significant (Supplementary Table S6).

Due to the statistically significant interactions between sex and nutritional knowledge (*p*_interaction_ < 0.001), as well as between dietary supplement use and participant age (*p_interaction_* = 0.001), two stratified analyses were conducted to explore the modifying effects of sex and dietary supplement use. Among men (*n* = 452), those with high self-assessed knowledge exhibited strongly and significantly higher odds of Intentional-VFF use (OR: 3.16, 95% CI: 1.70–5.90) compared to the reference group with low knowledge. Conversely, no statistically significant association was observed among women (*n* = 1198), where the odds of VFF consumption did not substantially differ between those with high knowledge (OR = 1.15; 95% CI: 0.73–1.81) and those in the low-knowledge reference category (Supplementary Table S7). Additionally, among non-users of dietary supplements (*n* = 924), each 1-year increment in age was associated with 12% (95% CI: 5–18%; *p* = 0.001) lower odds of Intentional-VFF use, while no significant age-related association was observed among dietary supplement users (*n* = 726; Supplementary Table S8). Furthermore, distinct factors associated with Intentional-VFF use were identified among women and dietary supplement users, including socioeconomic status (OR: 1.35, 95% CI: 1.03–1.78, and OR: 1.66, 95% CI: 1.13–2.43, respectively) and health status (OR: 0.59, 95% CI: 0.40–0.88, and OR: 0.48, 95% CI: 0.30–0.79, respectively) (Supplementary Tables S7 and S8).

### 3.4. Food-Record-VFF Consumption Details

The analysis of respondents’ dietary records identified 51 different VFFs, which were categorised into 6 distinct groups (Table 4). These included 19 types of juices and beverages (including fruit juices, vegetable and fruit juices, fruit nectars and drinks, energy drinks); 12 types of cereal products (including flours, corn flakes, flavoured breakfast cereals, oatmeal, rice and milk porridge); 7 types of sweets (including bars, candies, cookies); 6 types of dairy products (including yogurt, kefir, homogenised cottage cheese, milk drink and dessert); 4 types of milk substitutes (including soy drinks, oat drinks, almond drinks); and 3 types of instant products (instant cocoa powder). Cereal products as well as juices and beverages were the most common Food-Record-VFF groups consumed by university students (41.3% and 37.1%, respectively). The daily portion size of fortified products consumed was not large, ranging from a median of 6 g for instant powdered products to 83 mL for juices and beverages. Calcium was the most frequently consumed nutrient with the VFF products (consumed with cereals, sweets, dairy products and their substitutes, instant cocoa), while vitamin A and iron were the least frequently consumed (Table 4).

The average contribution of VFFs to total micronutrient intake (including intake from conventional foods, fortified foods, and dietary supplements) among Food-Record-VFF consumers is presented in Supplementary Table S9. The average contribution of fortified products to total vitamin intake ranged from 5.0% for vitamin A to 20.2% for vitamin C, while for minerals it ranged from 2.9% for magnesium to 11.3% for iron.

### 3.5. DASH-Style Diet Adherence

The likelihood of Intentional-VFF use across quintiles of DASH-style diet components is presented in Table 5. Compared to university students in the lowest quintile of legume, seed, and nut consumption, those in the highest quintile had significantly lower odds of using Intentional-VFFs (OR: 0.67, 95% CI: 0.45–1.00). Conversely, for wholegrains, the trend was reversed; respondents in the highest quintile were more likely to use fortified products than those in the lowest quintile (OR: 1.52, 95% CI: 1.04–2.22). No significant differences were found between the extreme quintiles for vegetables, fruits, low-fat dairy products, red and processed meat, sweetened beverage consumption, and sodium intake. However, each 50 g increment in vegetable consumption was associated with a 4% (95% CI: 1–7%; *p* = 0.013) lower odds of Intentional-VFF use. No statistically significant association was found between VFF usage and the DASH score.

## 4. Discussion

This study provides a detailed analysis of Intentional-VFF use among university students. It examines the underlying factors and motivations that influence their choices, quantifying their intake of these products and the corresponding micronutrient contributions.

This study reveals that the prevalence of Intentional-VFF use was over 40% among university students in Warsaw. The use of VFFs is a common dietary behaviour across various European populations, including the Netherlands [3], Portugal [9], the United Kingdom [25], and Ireland [7], where at least half of the adult population consumes such products. VFFs are more commonly consumed by younger individuals than by adults, primarily because many of these products are specifically designed to appeal to children and adolescents (e.g., flavoured breakfast cereals, sweetened beverages, and confectionery). In our previous study conducted in primary and secondary schools in Poland, we found that 71% of children and 64% of school-aged adolescents consumed these products [10]. Moreover, VFF consumption depends on factors such as product availability in the food market and regional dietary patterns.

In our study, over one-fifth of respondents were unsure or unaware whether they used fortified foods. The comparison with food records further suggested a potential discrepancy between participants’ awareness or ability to identify VFF use and their actual VFF consumption. A similar finding was reported in the Japanese population, where approximately one-third of participants were unaware of using fortified products and/or dietary supplements [26]. The food market is constantly developing products with added vitamins and minerals, which may lead to unconscious consumption. This is particularly true, as food choices are often influenced by factors beyond fortification, such as taste, convenience, or price [27]. In the present study, Intentional-VFF users considered the beneficial effects on health and the limited availability of nutrients in the diet as the main reasons for choosing these products. Similarly to our findings, in the Japanese population [26], poor nutritional balance or undernutrition is the main reason for using fortified products. The high proportion of respondents who were unaware or uncertain about their VFF consumption highlights a need to increase young adults’ awareness of the presence and nutritional composition of VFFs.

Our study provides valuable insights into the factors associated with the use of VFFs in university students. Those who intentionally used VFFs were younger, more likely to be female, had higher nutritional knowledge, made health-oriented dietary modifications, and reported using dietary supplements. Several studies reported a positive association between the likelihood of using certain VFFs and various demographic and lifestyle factors. These include younger age [8,9], female sex [9], higher educational attainment [9,28], regular physical activity [9], the presence of diet-related health concerns [28], non-smoking status [29], and the use of dietary supplements [28]. We also found that underweight university students were less likely to use these products than those with a normal BMI, possibly because they tend to consume less food overall. Studies conducted in Portugal also identified an association between VFF use and BMI levels [9]. However, the results indicated that individuals with obesity were less likely to consume these products compared to those with normal BMI. This may be partly explained by the fact that individuals with a higher BMI are more likely to underreport their food intake. Statistical significance should be distinguished from practical and public health significance. Although some associations were modest in magnitude, even small effects may potentially be relevant at the population level, particularly when exposure is common.

Stratified analyses showed important effect modifications that suggest even more cautious interpretation and generalisability of the overall association. Firstly, high nutritional knowledge was strongly associated with Intentional-VFF use only in men, which suggests distinct behavioral drivers between sexes. Secondly, an inverse association between age and Intentional-VFF use was observed among non-users of dietary supplements, whereas no significant age-related association was revealed among dietary supplement users. This may suggest that younger students who do not use dietary supplements may intentionally or unintentionally use VFFs as an alternative strategy to support their micronutrient intake. Therefore, given the heterogeneity of the observed associations, the results should be interpreted with caution, and further studies are needed to assess their generalisability and practical implications. Especially, future public health initiatives should not treat university students as a homogenous group, but rather tailor educational initiatives based on sex and supplement-use patterns.

In our study, the most commonly consumed VFF products were cereal products, followed by juices and beverages. This consumption pattern aligns with trends observed in other European countries. For instance, in the Netherlands, commonly consumed VFFs include fats and oils, non-alcoholic beverages, as well as dairy products and their substitutes [3]; in Finland, yogurt and fruit drinks are their predominant sources [8]; while in Portugal and Ireland, breakfast cereals and fat spreads are among the most frequently consumed fortified products [7,9]. In Poland, fat spreads are mandatorily fortified with vitamins A and D and were therefore not considered VFFs in our analysis.

In the present study, the usual intake of VFFs was low, with vitamins and minerals contributing an average of up to 20% and 11%, respectively. Similar findings have been reported in other European countries. In the UK [25] and Ireland [7], the contribution of fortified products to vitamin and mineral intake was no greater than 27% and 21%, respectively. In our study, vitamin C and pyridoxine accounted for the highest contributions to micronutrient intake, at approximately 20% each. In contrast, the most significant contributions to total intake in the UK and Ireland were observed for folates (15% and 27%, respectively) and iron (13% and 21%, respectively) [7,25]. In Ireland, it has consistently been found that the practice of VFF has enhanced protection against neural tube defect-affected pregnancies by increasing the consumption of folic acid-fortified products (mainly bread, milk, and fat spreads) among women of reproductive age [7]. However, in our previous study involving part of the same group of women, intake of folic acid from fortified products (mainly juices and beverages, as well as breakfast cereals) did not adequately compensate for inadequate dietary intake [14].

While the inclusion of VFFs in the diet may enhance micronutrient intake, it may also be associated with higher intakes of sugar, salt, and fat, as well as lower fibre content. On the Warsaw market, nearly 80% of mineral-fortified products were found to contain high levels of sugar and/or salt (as indicated on product labels), especially in cereal products, dairy products, and confectionery [30]. Therefore, in our study, we conducted a detailed assessment of respondents’ diets, with particular emphasis on adherence to the DASH diet. Although we did not observe an association between Intentional-VFF use and adherence to the DASH-style diet, some specific associations were observed for DASH-style diet components. Individuals in the highest quintile of legume, seed, and nut consumption had lower odds of VFF use than those in the lowest quintile. Conversely, respondents in the highest wholegrain consumption category had higher odds of VFF use than those in the lowest consumption category. In addition, when vegetable consumption was analysed as a continuous variable, each 50 g increment was associated with 4% lower odds of VFF use. The component-specific findings should be interpreted cautiously, as these analyses were exploratory and involved multiple comparisons. Different findings were reported in studies of the adult populations in Finland and Ireland, where consumption of fortified products was a marker of higher diet quality, reflected by greater fruit and vegetable intake in Finland [8] and greater fruit intake in Ireland [29]. It is possible that the choice of fortified products does not have a direct relation to diet quality, although further research is needed.

Among the strengths of this study are its large sample size, substantial representation of VFF users, and comprehensive characterisation of respondents based on socio-demographic and lifestyle factors. Moreover, detailed dietary intake information was collected from a large subset of participants using a 5-day food diary. Qualified dietitians or nutrition experts meticulously reviewed the returned questionnaires and supplemented any incomplete or ambiguous details regarding food intake. Participant recruitment was conducted continuously throughout the study period, and dietary records were therefore collected across different seasons and periods of the academic year, allowing potential seasonal variation in dietary habits to be represented in the study sample. This enabled us to develop logistic regression models to identify factors significantly associated with Intentional-VFF use, including adherence to a DASH-style diet.

The study has several limitations. First, its cross-sectional design precludes causal inference and limits the ability to determine the directionality of observed associations. Second, the extended data collection period (6 years), during which the introduction of new VFFs to the market may have influenced consumption patterns, potentially leading to the omission of certain products from the analysis. Some food consumption data were collected online during the 2020 COVID-19 lockdown, which may have affected dietary patterns; however, these records accounted for only 5% of the study sample and sensitivity analysis confirmed that their inclusion did not alter the overall findings. Third, many participants were uncertain about their use of VFFs, suggesting the presence of unconscious users, which could lead to an underestimation of the true prevalence of VFF use. Fourth, the analysis included a limited set of factors potentially influencing VFF use. Data on several potentially relevant determinants—such as the students’ educational field, living arrangements, and food purchasing responsibility—were not included. Furthermore, while we adjusted for general socioeconomic status, variables like precise family income and parental dietary habits were omitted, as our focus was on the independent behaviours of adult students. However, because family-of-origin factors continue to influence young adults’ dietary choices [31,32], unmeasured or residual confounding cannot be ruled out. These overlooked factors could share variance with included variables, potentially artificially inflating or attenuating the estimated odds ratios for the observed associations. A further limitation is the reliance on self-assessed, rather than objectively measured, nutritional knowledge. Because individuals frequently overestimate their actual competence [33], this variable is susceptible to information bias. Therefore, the positive association between nutritional knowledge and VFF use reflects perceived expertise and should be interpreted with caution. Finally, the voluntary (non-randomised) selection of study participants constitutes another limitation, as individuals adhering to healthier diets were more likely to return questionnaires and participate, potentially biasing the results. The voluntary recruitment of students from public universities in Warsaw may also limit the external validity of the findings, as the study population may not be representative of the broader young adult population.

## 5. Conclusions

Among university students, Intentional-VFF use was generally associated with younger age, female sex, higher nutritional knowledge, health-oriented dietary modifications, and the use of dietary supplements. Underweight participants had lower odds of Intentional-VFF use than those with a normal BMI, with no significant differences observed for overweight or obesity. However, the associations showed effect modification: the positive association between higher nutritional knowledge and Intentional-VFF use was limited to men, while the inverse association between age and Intentional-VFF use was evident only among dietary supplement non-users. Although these findings should be interpreted strictly as observed associations and do not imply causal relationships, they highlight the potential of targeted consumer profiling. Leveraging socio-demographic and lifestyle factors could help shape voluntary fortification strategies and guide the food industry toward the development of nutritionally balanced products.

No clear relationship between Intentional-VFF use and adherence to the DASH diet emerged, although a specific inverse association with vegetable consumption was observed. These results may support the development of nutrition education initiatives at universities aimed at increasing students’ awareness of the nutritional composition of VFFs and supporting more informed food choices. Additionally, future prospective cohort studies are needed to clarify the temporal relationships between these factors and VFF consumption, as well as to assess how evolving dietary behaviours influence VFF use over time.

## Figures and Tables

**Figure 1:**
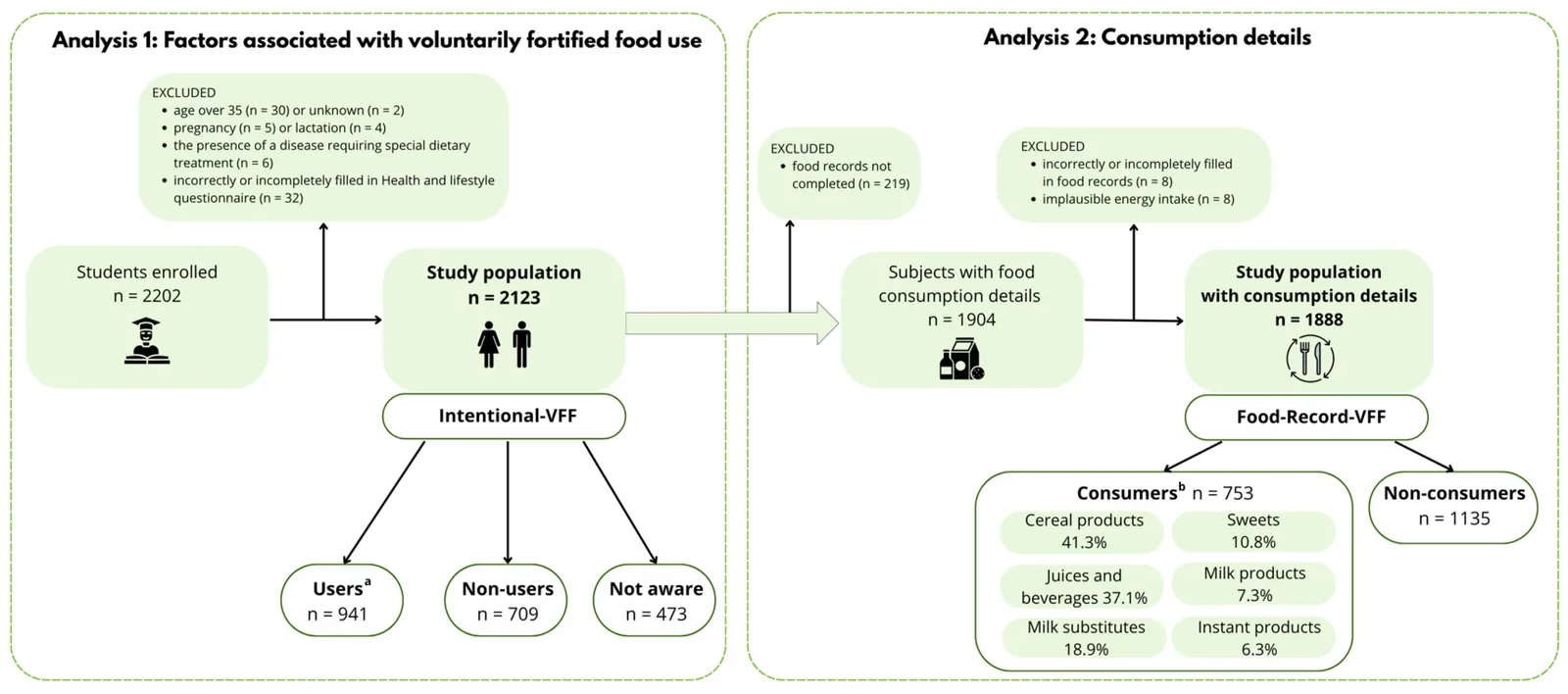
Flow chart of the study population, created with Canva (https://www.canva.com/; accessed on 24 September 2026). VFF—voluntarily fortified food. ^a^ Intentional-VFF user was considered an individual who indicated usage of VFF in the *Health and lifestyle questionnaire*; ^b^ Food-Record-VFF consumer was considered an individual who listed at least one specified food item in the *5-day food records*.

**Table 1:** Socio-demographic and lifestyle parameters stratified by intentional use of voluntarily fortified food (Intentional-VFF) in university students (*n* = 2123).

Parameters	Users ^a^ *n* = 941	Non-Users *n* = 709	Unaware ^b^ *n* = 473	*p*-Value ^c^
Age (mean ± SD, years)	21.8 ± 2.2	22.2 ± 2.3	21.9 ± 2.4	<0.001
Sex, %				
Female	78.0	65.4	66.6	<0.001
Male	22.0	34.6	33.4	
Place of residence, % ^d^				
Village	16.9	17.1	21.6	0.710
Small town	17.5	16.8	17.6	
City	56.8	59.4	53.1	
Socioeconomic status, %				
Very good or good	68.4	71.1	64.9	0.247
Average or poor	31.6	28.9	35.1	
Physical activity, %				
Low	39.4	40.9	50.5	0.490
Moderate	37.1	38.1	30.7	
High	23.5	21.0	18.8	
Body mass index (kg/m^2^)				
<18.5	6.9	9.3	8.3	0.078
18.5–24.9	74.8	70.2	71.5	
≥25	18.3	20.5	20.3	
Health status, %				
At least good	89.1	88.0	88.2	0.509
Average or poor	10.9	12.0	11.8	
Cigarette smoking, %				
No	85.4	82.2	76.7	0.078
Yes	14.6	17.8	23.3	
Self-assessment of nutritional knowledge, %				
Low	8.5	16.9	25.8	<0.001
Average	44.2	44.7	48.8	
High	47.3	38.4	25.4	
Diet modification, %				
No	44.1	60.1	58.6	<0.001
Exclusion or inclusion of certain foods	31.0	27.2	29.6	
Simultaneous exclusion and inclusion of certain foods	24.9	12.7	11.8	
Use of dietary supplements, %				
No	48.7	65.7	60.3	<0.001
Yes	51.3	34.3	39.7	

SD—standard deviation. ^a^ Intentional-VFF user was considered an individual who indicated usage of VFF in the *Health and lifestyle questionnaire*; ^b^ respondents who were unaware of VFF use were excluded from statistical analysis; ^c^ differences in distribution between VFF users vs. non-users were examined by the Mann–Whitney U test for quantitative variables and the Chi-squared test for categorical variables; ^d^ missing data on place of residence (7.0%).

**Table 2:** Reasons for using or not using voluntarily fortified foods (VFFs) by university students (*n* = 1650).

Parameters	Users ^a,b^	Non-Users
	*n* = 941	*n* = 709
Paying attention to whether the product is fortified, % ^c,d^		
No	25.8	65.6
Yes	60.1	15.0
Reason for including VFFs, % ^e^		
Beneficial effect on health	71.8	
Diet contains too few of the ingredients which the product is fortified with	29.7	
Taste preferences	28.8	
Doctor/pharmacist recommendation	3.1	
Other	5.5	
Reason for avoiding VFFs, % ^e^		
Eating properly and no need for such products		48.0
Never heard about such products		14.9
Lack of impact on health		11.9
Too expensive		10.7
Bad taste		8.2
Other		12.8

^a^ Intentional-VFF user was considered an individual who indicated usage of VFF in the *Health and lifestyle questionnaire*; ^b^ respondents who were unaware of VFF use were excluded from statistical analysis; ^c^ statistically significant differences between users and non-users at the level of *p*-value < 0.001 determined by Chi-square test; ^d^ the percentages do not add up to 100% due to missing data on paying attention to whether the product is fortified (16.4%); ^e^ each respondent could select one or more answers.

**Table 3:** Logistic regression of intentional use of voluntarily fortified food (Intentional-VFF) by socio-demographic and lifestyle factors in university students (*n* = 1650).

Study Factors	Age-Adjusted ^a,b^ OR (95% CI)	Multivariate-Adjusted ^a,b^ OR (95% CI)
Age (years)	0.92 (0.88–0.96)	0.94 (0.89–0.98)
Sex		
Female	1.00	1.00
Male	0.56 (0.45–0.70)	0.63 (0.49–0.81)
Place of residence		
Village	1.00	1.00
Small town	0.98 (0.70–1.36)	1.00 (0.71–1.41)
City	0.89 (0.68–1.16)	0.85 (0.64–1.12)
Missing data ^c^	0.97 (0.62–1.50)	0.73 (0.46–1.17)
Socioeconomic status		
Very good or good	1.00	1.00
Average or poor	1.15 (0.93–1.43)	1.19 (0.95–1.50)
Physical activity		
Low	1.00	1.00
Moderate	1.00 (0.80–1.25)	0.93 (0.73–1.18)
High	1.15 (0.89–1.49)	1.03 (0.76–1.38)
Body mass index (kg/m^2^)		
<18.5	0.68 (0.47–0.98)	0.63 (0.43–0.92)
18.5–24.9	1.00	1.00
≥25	0.89 (0.69–1.14)	1.08 (0.83–1.42)
Health status		
At least good	1.00	1.00
Average or poor	0.89 (0.66–1.21)	0.77 (0.55–1.08)
Cigarette smoking		
No	1.00	1.00
Yes	0.80 (0.61–1.04)	0.94 (0.71–1.24)
Self-assessment of nutritional knowledge		
Low	1.0	1.0
Average	1.88 (1.36–2.59)	1.53 (1.09–2.14)
High	2.39 (1.73–3.30)	1.70 (1.20–2.42)
Diet modification		
No	1.00	1.00
Exclusion or inclusion of certain foods	1.53 (1.22–1.92)	1.28 (1.01–1.63)
Simultaneous exclusion and inclusion of certain foods	2.65 (2.01–3.51)	2.09 (1.55–2.83)
Use of dietary supplements		
No	1.00	1.00
Yes	2.01 (1.65–2.46)	1.62 (1.30–2.01)

OR—odds ratio; CI—confidence interval. ^a^ Intentional-VFF user was considered an individual who indicated usage of VFF in the *Health and lifestyle questionnaire*; ^b^ respondents who were unaware of VFF use were excluded from statistical analysis; ^c^ missing data on the place of residence (7.0%) were modelled as a separate category.

**Table 4:** Median (P5–P95) serving size and intake of micronutrients provided by voluntarily fortified foods (VFFs) in Food-Record consumers’ diets (*n* = 753).

Serving Size and Nutrients Provided with VFF ^b^	Group of VFF Median (P5–P95) ^a^					
	Cereal Products	Juices and Beverages	Milk Substitutes	Sweets	Milk Products	Instant Products
	*n* = 311 (41.3%)	*n* = 279 (37.1%)	*n* = 142 (18.9%)	*n* = 81 (10.8%)	*n* = 55 (7.3%)	*n* = 47 (6.3%)
SERVING SIZE PER DAY, g or mL	13 (4–60)	83 (30–300)	67 (8–234)	10 (5–36)	46 (20–150)	6 (2–50)
Vitamin A, µg RE	–	13.5 (0.6–431)	–	–	–	–
Vitamin D, µg	–	–	0.52 (0.06–1.74)	–	0.21 (0.01–0.63)	0.25 (0.10–2.50)
Vitamin E, mg TE	–	0.12 (0.01–7.50)	–	–	–	–
Vitamin C, mg	6.80 (2.04–25.5)	22.4 (0.68–89.6)	–	0.10 (0.03–8.1)	–	–
Thiamine, mg	0.16 (0.04–0.60)	0.04 (0.01–0.35)	–	0.03 (0.01–0.17)	–	0.07 (0.01–0.40)
Riboflavin, mg	0.19 (0.06–0.74)	0.02 (0.01–0.81)	0.14 (0.02–0.51)	–	–	0.08 (0.01–0.41)
Niacin, mg	2.01 (0.58–7.74)	2.19 (0.03–21.1)	–	0.14 (0.04–1.41)	–	0.03 (0.01–2.0)
Pyridoxine, mg	0.23 (0.07–0.85)	0.55 (0.05–4.0)	–	–	0.03 (0.01–0.18)	0.10 (0.03–0.45)
Folic acid, µg DFE	23.8 (6.80–87.3)	47.9 (4.41–262)	–	4.1 (0.41–26.8)	–	0.44 (0.15–42.0)
Cobalamin, µg	0.11 (0.02–0.42)	0.19 (0.06–3.16)	0.26 (0.03–0.88)	–	–	–
Calcium, mg	3.20 (0.48–109)	–	82 (10.4–289)	7.2 (1.34–38.4)	57 (20–391)	1.6 (0.5–150)
Iron, mg	1.58 (0.27–6.05)	–	–	–	–	–
Magnesium, mg	–	–	–	9.0 (2.7–40)	–	4.3 (1.4–35)

RE—retinol equivalent; TE—α-tocopherol equivalent; DFE—dietary folate equivalent; P—percentile. ^a^ Food-Record-VFF consumer was considered an individual who listed at least one specified food item in the *5-day food records*; ^b^ vitamin or mineral most frequently supplied by VFF groups (presented when consumed by at least 10% of consumers).

**Table 5:** The likelihood of intentional use of voluntarily fortified foods (Intentional-VFFs) across quintiles of food consumption and DASH score in university students (*n* = 1473).

DASH Diet Components	Quintiles of Food Consumption Multivariate-Adjusted OR (95% CI) ^a,b,c^					Unit Per Day	OR (95% CI) Per Unit	*p*-Value
	1	2	3	4	5			
Vegetables	1.00	1.06 (0.75–1.51)	0.80 (0.56–1.14)	0.73 (0.51–1.05)	0.79 (0.53–1.16)	50 g	0.96 (0.93–0.99)	0.013
Fruits	1.00	1.04 (0.73–1.47)	1.27 (0.88–1.84)	0.95 (0.66–1.37)	1.24 (0.84–1.83)	50 g	1.02 (0.98–1.05)	0.363
Legumes, seeds, nuts	1.00	1.01 (0.70–1.45)	0.84 (0.60–1.19)	0.83 (0.58–1.19)	0.67 (0.45–1.00)	10 g	0.99 (0.96–1.03)	0.793
Wholegrains	1.00	1.35 (0.95–1.92)	1.25 (0.87–1.80)	1.34 (0.93–1.94)	1.52 (1.04–2.22)	25 g	1.06 (0.99–1.12)	0.063
Low-fat dairy products	1.00	1.30 (0.91–1.85)	1.30 (0.90–1.85)	1.38 (0.96–1.98)	1.27 (0.89–1.83)	25 g	1.01 (0.99–1.04)	0.386
Red and processed meat	1.00	0.79 (0.55–1.13)	0.72 (0.49–1.04)	0.72 (0.50–1.05)	0.78 (0.53–1.14)	25 g	0.97 (0.93–1.01)	0.126
Sweetened beverages	1.00 ^d^		2.56 (0.92–7.11)	0.88 (0.66–1.19)	0.96 (0.72–1.28)	25 g	1.00 (0.99–1.01)	0.594
Sodium ^e^	1.00	1.01 (0.70–1.43)	1.31 (0.90–1.89)	1.10 (0.76–1.59)	1.10 (0.75–1.63)	600 mg/1000 kcal	1.02 (0.86–1.21)	0.837
	**Quintiles of the DASH score, points ^a,b^**							
**Intentional-VFF use**	**<11** (*n* = 262)	**11–14** (*n* = 296)	**15–17** (*n* = 259)	**18–21** (*n* = 312)	**≥22** (*n* = 344)			
Yes (%)	53.2	57.2	57.9	59.9	59.8			0.479 ^f^
No (%)	46.8	42.8	42.1	40.1	40.1		–	
Multivariate-adjusted OR (95%CI)	1.00	1.16 (0.80–1.69)	1.04 (0.73–1.49)	1.02 (0.71–1.45)	0.95 (0.67–1.35)		1.01 (0.99–1.02)	0.647

DASH—Dietary Approaches to Stop Hypertension, OR—odds ratio, CI—confidence interval. ^a^ respondents who were unaware of VFF use were excluded from statistical analysis—the sample of 1473 represents the intersection of participants with valid food records (*n* = 1888), excluding those who were unaware of their VFF use (*n* = 415); ^b^ logistic regression models were adjusted for age, sex, Body Mass Index, nutritional knowledge, diet modification and use of dietary supplements; ^c^ mutually adjusted for food groups that constitute the DASH diet components; ^d^ a reference category includes the two lowest quintiles due to a lack of consumption of these products in the first and the second quintiles; ^e^ sodium intake only from food products and prepared meals (the respondent’s addition of salt to the meal at the table was not taken into account); ^f^ *p*-heterogeneity was calculated using the chi-squared test.

## Data Availability

The data presented in this study are available from the corresponding author. The data are stored within a highly secure institutional IT environment under the supervision of Ewa Sicińska (PI). Investigators may request access to the study’s deidentified data upon reasonable request to the PI.

## Supplementary Materials

The following supporting information can be downloaded at: https://www.mdpi.com/article/10.3390/nu18193188/s1, Supplementary Table S1. Sex-specific percentile cut-offs for DASH diet components and scoring criteria (*n* = 1473); Supplementary Table S2. Comparison of questionnaire-declared intentional voluntarily fortified food (Intentional-VFF) use and consumption recorded in food records (Food-Record-VFF) among participants with complete data from both methods (*n* = 1888); Supplementary Table S3. Diagnostic assessment of the main multivariable logistic regression model; Supplementary Table S4. Sensitivity analysis: logistic regression of intentional use of voluntarily fortified food (Intentional-VFF) by sociodemographic and lifestyle factors using complete-case data (*n* = 1538); Supplementary Table S5. Sensitivity analyses of intentional use of voluntarily fortified foods (Intentional-VFF) by sociodemographic and lifestyle factors: Model A excluding COVID-19 lockdown data (*n* = 1578) and Model B adjusting for recruitment year (*n* = 1650); Supplementary Table S6. Sensitivity analysis: logistic regression of voluntarily fortified food (VFF) use by sociodemographic and lifestyle factors after reclassification of participants uncertain about their VFF use as VFF users (*n* = 1803); Supplementary Table S7. Logistic regression analysis of intentional use of voluntarily fortified food (Intentional-VFF) by sociodemographic and lifestyle factors, stratified by sex (*n* = 1650); Supplementary Table S8. Logistic regression analysis of intentional use of voluntarily fortified food (Intentional-VFF) by sociodemographic and lifestyle factors, stratified by dietary supplement use (*n* = 1650); Supplementary Table S9. Contribution of voluntarily fortified foods (VFFs) to total micronutrient intake among Food–Record–VVF consumers (*n* = 753).
