# Supplementary material for: Intentional Use of Voluntarily Fortified Foods in Relation to Socio-Demographic and Lifestyle Factors, Including Adherence to a DASH-Style Diet, Among University Students
Source: Nutrients. 2026 Sep 26;18(19):3188. doi: 10.3390/nu18193188 (PMC13666850; doi:10.3390/nu18193188)
Supplement: Supplementary file 1 [file nutrients-18-03188-s001.zip › nutrients-4540145-supplementary.pdf]

SUPPLEMENTARY MATERIALS

Intentional Use of Voluntarily Fortified Foods in Relation to Socio-Demographic and Lifestyle Factors, Including Adherence to a DASH-Style Diet, Among University Students

Ewa Sicińska *, Dawid Madej, Maria Karolina Szmidt, Justyna Czerniak and Joanna Kaluza

Department of Human Nutrition, Institute of Human Nutrition Sciences, Warsaw University of Life Sciences (WULS-SGGW), Nowoursynowska 166, 02-787 Warsaw, Poland

* Correspondence: ewa_sicinska@sggw.edu.pl

Supplementary Table S1. Sex-specific percentile cut-offs for DASH diet components and scoring criteria (*n* = 1473)

DASH diet components	Women (*n*=1097)				Men (*n*=376)				Scoring criteria
	P20	P40	P60	P80	P20	P40	P60	P80	
Vegetables (g)	154	223	296	414	160	234	299	453	*Q1 = 0 points*<br>*Q2 = 1 point*<br>*Q3 = 2 points*<br>*Q4 = 3 points*<br>*Q5 = 4 points*
Fruits (g)	118	193	265	414	46	128	224	351	
Legumes, seeds, nuts (g)	0.6	7.2	17.8	39.1	0	1.9	11.7	30.6	
Wholegrains (g)	22.1	47.6	73.2	105	6.7	36.7	67.0	112	
Low-fat dairy products (g)	39.7	88.0	142	212	13.3	68.7	134	211	
Red and processed meat (g)	13.3	34.0	58.1	94.7	33.9	80.5	118	190	*Q1 = 4 points*<br>*Q2 = 3 points*<br>*Q3 = 2 points*<br>*Q4 = 1 point*<br>*Q5 = 0 points*
Sweetened beverages (g)	0	0	0	200	0	0	62.5	183	
Sodium (mg/1000 kcal)	1153	1375	1583	1828	1214	1443	1643	1910	

DASH - Dietary Approaches to Stop Hypertension, P – percentile.

Supplementary Table S2. Comparison of questionnaire-declared intentional voluntarily fortified food (Intentional-VFF) use and consumption recorded in food records (Food-Record-VFF) among participants with complete data from both methods (*n* = 1888)

	Food-Record-VFF consumption, *n* (%)		Total
	No	Yes	
Intentional-VFF use, *n* (%)			
No	405 (21.4%)	216 (11.4%)	621 (32.9%)
Yes	468 (24.8%)	384 (20.3%)	852 (45.1%)
Don’t know	262 (13.9%)	153 (8.1%)	415 (22.0%)
Total	1135 (60.1%)	753 (39.9%)	1888 (100%)

Supplementary Table S3. Diagnostic assessment of the main multivariable logistic regression model

Diagnostic domain	Method/statistics	Results
Multicollinearity assessment	Variance inflation factor (VIF)*	1.03–2.70
	Tolerance*	0.36–0.96
Goodness-of-fit	Hosmer–Lemeshow test	$\chi^2 = 8.23$, p = 0.412
	Pearson goodness-of-fit statistic	$\chi^2 = 1656.63$, df = 1632, χ^2/df = 1.015, p = 0.330
Discrimination measures	Area under the ROC curve (AUC)	0.659
	Standard error of AUC	0.0136
Influential observations	Cook's distance	Max ≈ 0.0056
Residual diagnostics	Standardised Pearson residuals	Max absolute value ≈ 2.41
	Standardised deviance residuals	Max absolute value ≈ 1.96
Interaction terms	Likelihood ratio (LR) tests	Significant interactions were identified for sex × nutritional knowledge ($p < 0.001$) and dietary supplement use × age ($p = 0.001$).

* range of values for all variables included in the multivariable logistic regression model.

Supplementary Table S4. Sensitivity analysis: logistic regression of intentional use of voluntarily fortified food (Intentional-VFF) by sociodemographic and lifestyle factors using complete-case data (n = 1538)

Study factors	Age–adjusted [a, b] OR (95% CI)	Multivariate–adjusted [a, b] OR (95% CI)
Age (years)	0.92 (0.88–0.97)	0.94 (0.89–0.99)
Sex		
Female	1.00	1.00
Male	0.56 (0.44–0.70)	0.64 (0.50–0.84)
Place of residence		
Village	1.00	1.00
Small town	0.98 (0.70–1.36)	0.99 (0.70–1.40)
City	0.89 (0.68–1.16)	0.84 (0.64–1.11)
Socioeconomic status		
Very good or good	1.00	1.00
Average or poor	1.18 (0.95–1.48)	1.22 (0.96–1.55)
Physical activity		
Low	1.00	1.00
Moderate	1.01 (0.80–1.26)	0.94 (0.73–1.19)
High	1.15 (0.88–1.50)	1.01 (0.74–1.37)
Body mass index (kg/m^2)		
< 18.5	0.68 (0.47–0.99)	0.63 (0.43–0.94)
18.5 – 24.9	1.00	1.00
≥ 25	0.85 (0.66–1.10)	1.03 (0.78–1.37)
Health status		
At least good	1.00	1.00
Average or poor	0.93 (0.67–1.27)	0.81 (0.57–1.14)
Cigarette smoking		
No	1.00	1.00
Yes	0.79 (0.60–1.04)	0.94 (0.70–1.25)
Self–assessment of nutritional knowledge		
Low	1.0	1.0
Average	1.91 (1.38–2.65)	1.56 (1.11–2.21)
High	2.55 (1.83–3.55)	1.82 (1.27–2.61)
Diet modification		
No	1.00	1.00
Exclusion or inclusion of certain foods	1.60 (1.26–2.02)	1.32 (1.03–1.70)
Simultaneous exclusion and inclusion of certain foods	2.61 (1.95–3.50)	1.98 (1.44–2.71)
Use of dietary supplements		
No	1.00	1.00
Yes	2.02 (1.64–2.49)	1.59 (1.27–2.00)

OR – odds ratio; CI – confidence interval. [a] Intentional-VFF user was considered an individual who indicated usage of VFF in the *Health and lifestyle questionnaire*; [b] respondents who were unaware of VFF use were excluded from statistical analysis.

Supplementary Table S5. Sensitivity analyses of intentional use of voluntarily fortified foods (Intentional-VFF) by sociodemographic and lifestyle factors: Model A excluding COVID-19 lockdown data (n = 1578) and Model B adjusting for recruitment year (n = 1650).

Study factors	Model A Excluding COVID-19 lockdown data	Model B Adjusting for recruitment year
	Multivariate–adjusted [a, b] OR (95% CI)	Multivariate–adjusted [a, b] OR (95% CI)
Age (years)	0.95 (0.90–0.99)	0.94 (0.89–0.98)
Sex		
Female	1.00	1.00
Male	0.62 (0.48–0.81)	0.63 (0.49–0.82)
Place of residence		
Village	1.00	1.00
Small town	1.02 (0.71–1.46)	1.00 (0.71–1.41)
City	0.82 (0.61–1.09)	0.85 (0.64–1.12)
Socioeconomic status		
Very good or good	1.00	1.00
Average or poor	1.22 (0.96–1.54)	1.19 (0.95–1.50)
Physical activity		
Low	1.00	1.00
Moderate	0.88 (0.69–1.12)	0.93 (0.73–1.17)
High	0.96 (0.71–1.30)	1.02 (0.76–1.37)
Body mass index (kg/m^2)		
< 18.5	0.66 (0.45–0.96)	0.63 (0.43–0.92)
18.5 – 24.9	1.00	1.00
≥ 25	1.12 (0.84–1.47)	1.08 (0.83–1.42)
Health status		
At least good	1.00	1.00
Average or poor	0.73 (0.52–1.02)	0.77 (0.55–1.08)
Cigarette smoking		
No	1.00	1.00
Yes	0.94 (0.71–1.26)	0.94 (0.71–1.25)
Self–assessment of nutritional knowledge		
Low	1.0	1.0
Average	1.59 (1.12–2.25)	1.53 (1.09–2.14)
High	1.73 (1.21–2.48)	1.70 (1.20–2.41)
Diet modification		
No	1.00	1.00
Exclusion or inclusion of certain foods	1.24 (0.97–1.59)	1.28 (1.01–1.62)
Simultaneous exclusion and inclusion of certain foods	2.20 (1.61–3.00)	2.08 (1.54–2.82)
Use of dietary supplements		
No	1.00	1.00
Yes	1.57 (1.25–1.96)	1.62 (1.30–2.02)
Data collection (years)[c]	-	0.98 (0.92–1.05)

OR – odds ratio; CI – confidence interval. [a] Intentional-VFF user was considered an individual who indicated usage of VFF in the *Health and lifestyle questionnaire*; [b] respondents who were unaware of VFF use were excluded from statistical analysis; [c] annual recruitment proportions for the study population were as follows: 2017 (28.5%), 2018 (20.2%), 2019 (18.4%), 2020 (11.7%), 2021 (7.8%), and 2022 (13.4%).

Supplementary Table S6. Sensitivity analysis: logistic regression of voluntarily fortified food (VFF) use by sociodemographic and lifestyle factors after reclassification of participants uncertain about their VFF use as VFF users (n = 1803)*

Study factors	Age–adjusted [a, b] OR (95% CI)	Multivariate–adjusted [a, b] OR (95% CI)
Age (years)	0.92 (0.88–0.96)	0.94 (0.90–0.98)
Sex		
Female	1.00	1.00
Male	0.59 (0.48–0.73)	0.63 (0.49–0.80)
Place of residence		
Village	1.00	1.00
Small town	0.96 (0.70–1.33)	1.01 (0.73–1.41)
City	0.86 (0.66–1.11)	0.86 (0.66–1.12)
Socioeconomic status		
Very good or good	1.00	1.00
Average or poor	1.13 (0.92–1.40)	1.17 (0.94–1.46)
Physical activity		
Low	1.00	1.00
Moderate	0.94 (0.76–1.16)	0.89 (0.71–1.12)
High	1.07 (0.83–1.38)	1.01 (0.76–1.34)
Body mass index (kg/m^2)		
< 18.5	0.71 (0.51–1.01)	0.66 (0.46–0.95)
18.5 – 24.9	1.00	1.00
≥ 25	0.89 (0.70–1.13)	1.06 (0.82–1.37)
Health status		
At least good	1.00	1.00
Average or poor	0.88 (0.65–1.19)	0.75 (0.54–1.03)
Cigarette smoking		
No	1.00	1.00
Yes	0.91 (0.70–1.17)	1.05 (0.81–1.37)
Self–assessment of nutritional knowledge		
Low	1.0	1.0
Average	1.42 (1.06–1.90)	1.16 (0.85–1.57)
High	1.62 (1.21–2.18)	1.17 (0.85–1.61)
Diet modification		
No	1.00	1.00
Exclusion or inclusion of certain foods	1.42 (1.14–1.77)	1.26 (1.00–1.59)
Simultaneous exclusion and inclusion of certain foods	2.29 (1.74–3.02)	1.93 (1.44–2.60)
Use of dietary supplements		
No	1.00	1.00
Yes	1.86 (1.53–2.26)	1.58 (1.28–1.95)

OR – odds ratio; CI – confidence interval. [a] VFF users were defined as individuals who reported Intentional-VFF use in the *Health and Lifestyle Questionnaire*, as well as participants who were uncertain about their VFF use ('don't know') but were classified as Food-Record-VFF consumers; [b] the remaining participants who were uncertain about their VFF use were excluded from the statistical analysis. * VFF use: 1094 (60.7%).

Supplementary Table S7. Logistic regression analysis of intentional use of voluntarily fortified food (Intentional-VFF) by sociodemographic and lifestyle factors, stratified by sex (n = 1650)[*]

Study factors	Women (n = 1198) Multivariate–adjusted [a, b] OR (95% CI)	Men (n = 452) Multivariate–adjusted [a, b] OR (95% CI)
Age (years)	0.93 (0.88–0.99)	0.95 (0.87–1.05)
Place of residence		
Village	1.00	1.00
Small town	0.97 (0.65–1.45)	1.21 (0.59–2.49)
City	0.85 (0.62–1.18)	0.96 (0.53–1.77)
Socioeconomic status		
Very good or good	1.00	1.00
Average or poor	1.35 (1.03–1.78)	0.90 (0.57–1.41)
Physical activity		
Low	1.00	1.00
Moderate	1.00 (0.77–1.31)	0.89 (0.52–1.54)
High	1.02 (0.71–1.48)	0.97 (0.56–1.68)
Body mass index (kg/m^2)		
< 18.5	0.58 (0.40–0.86)	2.20 (0.51–9.52)
18.5 – 24.9	1.00	1.00
≥ 25	0.90 (0.63–1.28)	1.39 (0.89–2.18)
Health status		
At least good	1.00	1.00
Average or poor	0.59 (0.40–0.88)	1.51 (0.79–2.89)
Cigarette smoking		
No	1.00	1.00
Yes	1.06 (0.75–1.49)	0.72 (0.43–1.20)
Self–assessment of nutritional knowledge		
Low	1.0	1.0
Average	1.23 (0.79–1.91)	1.67 (0.93–2.99)
High	1.15 (0.73–1.81)	3.16 (1.70–5.90)
Diet modification		
No	1.00	1.00
Exclusion or inclusion of certain foods	1.16 (0.88–1.54)	1.74 (1.06–2.87)
Simultaneous exclusion and inclusion of certain foods	2.13 (1.51–3.02)	1.87 (0.99–3.53)
Use of dietary supplements		
No	1.00	1.00
Yes	1.51 (1.17–1.94)	2.15 (1.35–3.41)

OR – odds ratio; CI – confidence interval. [a] Intentional-VFF user was considered an individual who indicated usage of VFF in the *Health and lifestyle questionnaire*; [b] respondents who were unaware of VFF use were excluded from statistical analysis. [*] Intentional-VFF use: women – 734 (61.3%); men – 207 (45.8%).

Supplementary Table S8. Logistic regression analysis of intentional use of voluntarily fortified food (Intentional-VFF) by sociodemographic and lifestyle factors, stratified by dietary supplement use (n = 1650)*

Study factors	Supplement users (n = 726)<br>Multivariate–adjusted [a, b] OR (95% CI)	Non-users (n = 924)<br>Multivariate–adjusted [a, b] OR (95% CI)
Age (years)	0.99 (0.93–1.06)	0.88 (0.82–0.95)
Sex		
Female	1.00	1.00
Male	0.89 (0.58–1.37)	0.51 (0.36–0.71)
Place of residence		
Village	1.00	1.00
Small town	1.03 (0.59–1.78)	0.99 (0.63–1.57)
City	0.87 (0.56–1.37)	0.85 (0.59–1.21)
Socioeconomic status		
Very good or good	1.00	1.00
Average or poor	1.66 (1.13–2.43)	1.02 (0.76–1.38)
Physical activity		
Low	1.00	1.00
Moderate	0.80 (0.54–1.16)	1.08 (0.79–1.47)
High	0.94 (0.61–1.46)	1.17 (0.77–1.78)
Body mass index (kg/m^2)		
< 18.5	0.62 (0.37–1.07)	0.64 (0.37–1.10)
18.5 – 24.9	1.00	1.00
≥ 25	0.79 (0.51–1.22)	1.31 (0.92–1.87)
Health status		
At least good	1.00	1.00
Average or poor	0.48 (0.30–0.79)	1.13 (0.71–1.80)
Cigarette smoking		
No	1.00	1.00
Yes	1.04 (0.64–1.69)	0.88 (0.62–1.26)
Self–assessment of nutritional knowledge		
Low	1.0	1.0
Average	1.21 (0.62–2.35)	1.56 (1.03–2.35)
High	1.05 (0.54–2.04)	2.13 (1.37–3.31)
Diet modification		
No	1.00	1.00
Exclusion or inclusion of certain foods	0.96 (0.66–1.40)	1.46 (1.06–2.01)
Simultaneous exclusion and inclusion of certain foods	1.58 (1.04–2.40)	2.79 (1.76–4.44)

OR – odds ratio; CI – confidence interval. [a] Intentional-VFF user was considered an individual who indicated usage of VFF in the *Health and lifestyle questionnaire*; [b] respondents who were unaware of VFF use were excluded from statistical analysis. * Intentional-VFF use: supplement users – 483 (66.5%), non-users – 458 (49.6%).

Supplementary Table S9. Contribution of voluntarily fortified foods (VFFs) to total micronutrient intake among Food–Record–VVF consumers (n = 753)

Nutrients	Number of consumers [a, b]	Total diet intake [c]	VFF intake [c]				Average contribution of VFFs to the total diet [c]
	n (%)	Mean ± SD	Mean ± SD	P5	P50	P95	%
Vitamin A, µg RE	363 (48.2)	1213 ± 1074	62.4 ± 151	0.45	13.5	326	5.0
Vitamin D, µg	293 (38.9)	23.3 ± 102	0.33 ± 0.59	0.03	0.33	1.60	13.3
Vitamin E, mg TE	612 (81.3)	18.3 ± 42.7	3.72 ± 21.2	0.01	0.10	2.89	6.4
Vitamin C, mg	424 (56.3)	171 ± 347	23.0 ± 48.1	1.20	11.6	74.7	20.2
Thiamine, mg	623 (82.7)	1.45 ± 1.88	0.17 ± 0.24	0.01	0.10	0.60	12.3
Riboflavin, mg	649 (86.2)	1.97 ± 1.25	0.23 ± 0.29	0.01	0.14	0.71	11.4
Niacin, mg	642 (85.3)	21.2 ± 28.7	2.69 ± 3.97	0.05	1.26	10.3	12.1
Pyridoxine, mg	617 (81.9)	2.90 ± 3.19	0.67 ± 0.99	0.03	0.31	2.66	20.0
Folate, µg DFE	576 (76.5)	354 ± 203	48.3 ± 70.4	2.41	28.3	159	13.4
Cobalamin, µg	629 (83.5)	10.8 ± 44.8	0.45 ± 0.73	0.04	0.21	1.67	10.7
Calcium, mg	642 (85.3)	805 ± 332	56.6 ± 95.9	0.70	22.2	225	6.9
Iron, mg	577 (76.3)	14.2 ± 9.67	1.55 ± 2.00	0.05	0.80	5.15	11.3
Magnesium, mg	583 (77.4)	339 ± 129	9.64 ± 15.4	0.36	5.36	30.6	2.9

VFF – voluntarily fortified food; SD – standard deviation; P – percentile; RE – retinol equivalent; TE – α-tocopherol equivalent; DFE – dietary folate equivalent.

[a] Food–Record–VFF consumer was considered an individual who listed at least one specified food item in the *5-day food records*; [b] percentage of respondents calculated in relation to all Food–Record–VFF consumers; [c] data calculated in relation to those who consumed a specific micronutrient from VFFs.
